# Adult Day Services as a Platform for Innovation: Moving Beyond Simply Attendance

**DOI:** 10.1093/geroni/igaa057.2101

**Published:** 2020-12-16

**Authors:** Keith Anderson

**Affiliations:** University of Texas at Arlington, Arlington, Texas, United States

## Abstract

At the most basic level, adult day services (ADS) provide a congregate environment for participants and respite for caregivers. Researchers often evaluate the impact of ADS on participants and caregivers in terms of attendance; however, what happens in ADS (e.g., specific programs and interventions) may be equally or even more important than simply attendance. In this presentation, we review four recent innovative studies conducted in the ADS setting with participants: (a) a board game intervention to improve cognitive functioning; (b) a cognitive behavioral intervention to improve sleep; (c) an aromatherapy intervention to address behavioral issues; and (d) a dance and movement intervention to stimulate physical activity. While these interventions had varying levels of effectiveness, they do support a growing body of evidence that ADS can serve as a platform for innovation and suggest that attendance may be simply one facet of the overall ADS experience.

